# The value of total tumor diameter in unilateral multifocal papillary thyroid carcinoma: a propensity score matching analysis

**DOI:** 10.3389/fendo.2023.1217613

**Published:** 2023-09-06

**Authors:** Zhu-juan Wu, Bao-ying Xia, Zi-wei Chen, Hao Gong, Munire Abuduwaili, Zhi-chao Xing, An-ping Su

**Affiliations:** ^1^ Department of Thyroid & Parathyroid Surgery, West China Hospital, Sichuan University, Chengdu, China; ^2^ Department of Surgery, The University of Hong Kong-Shenzhen Hospital, Shenzhen, China

**Keywords:** papillary thyroid carcinoma, propensity score matching analysis, total tumor diameter, unilateral multifocal, lymph node metastases

## Abstract

**Background:**

Tumor multifocality is frequently observed in papillary thyroid carcinoma (PTC). However, the maximum tumor diameter (MTD), currently utilized in various staging schemes, might not accurately indicate the level of aggressiveness exhibited by multifocal tumors. We aimed to investigate the relationship between total tumor diameter (TTD) and clinicopathological features of papillary thyroid carcinoma.

**Methods:**

Retrospective data analysis was done on 1936 individuals who underwent complete thyroidectomy for PTC. Patients were classified into subgroups according to unilateral multifocality, central lymph node metastasis (CLNM) and lateral lymph node metastasis (LLNM). The relationships of clinicopathological features among these groups were analyzed.

**Results:**

Unilateral multifocality was observed in 117 patients. The clinicopathological features of the unilateral multifocal PTC were similar to the unifocal PTC with approximate TTD. The unilateral multifocality played no independent role in CLNM and LLNM. Moreover, the efficiency of TTD in predicting CLNM and LLNM was significantly higher than that of MTD.

**Conclusion:**

In the case of unilateral multifocal PTC, TTD is a more accurate indicator of the biological characteristics of the tumor than MTD.

## Introduction

Thyroid cancer is the ninth most common cancer worldwide, and its prevalence has increased significantly over the last 40 years (1). In 2020, there were 586,202 new cases of thyroid cancer, with papillary thyroid carcinoma (PTC) accounting for more than 80% of all thyroid cancers (1). In general, PTC is regarded as a disease with a favorable prognosis. However, some PTCs with specific clinicopathological characteristics are aggressive and have poor prognoses (2). Tumor multifocality is a frequent observation in PTC, occurring in 18–87% of documented cases (3). Multifocality in thyroid cancer is the concurrent existence of multiple tumor foci within the thyroid gland (4). Depending on where the tumors are, the meaning of multifocality can be broken down into unilateral multifocality (two or more lesions in the same lobe), bilateral multifocality (two or more lesions in the same lobe and one or more lesions in the opposite lobe), or bilaterality (one lesion in each lobe). And a report showed that unilateral multifocality and bilaterality could be two different multifocal entities in patients with PTC (5).

Multiple studies have shown that multifocality is associated with high-risk characteristics of PTC, such as an aggressive histologic subtype, extrathyroidal extension, lymph node involvement, distant metastasis, and recurrence (6, 7). However, the current staging system still defines the size of multifocal tumors in the same manner as that of unifocal tumors, concentrating on the maximum tumor diameter (MTD) (8). In recent years, many studies have also reported the concept of total tumor diameter (TTD) (9–11). Some demonstrated that TTD could reflect a tumor’s biological characteristics and aggressiveness more accurately than MTD (12).

However, the previous researchers took the unilateral multifocality and bilaterality as the same multifocality in their studies (5, 11). The purpose of the present study was to evaluate the association between TTD and unilateral multifocal PTC using the propensity score matching (PSM) method to provide an updated and more comprehensive assessment of the risk for unilateral multifocal PTC.

## Methods

### Patients

Data for pathologically confirmed PTC patients who underwent thyroid surgery in the Department of Thyroid Surgery, West China Hospital (Chengdu, China) between 2013 and 2018 were collected and reviewed. 2322 PTC Patients who were > 18 years old and underwent the first-time total thyroidectomy with central lymph nodes dissection were enrolled. After excluding PTC with bilateral multifocality (260 cases,11.2%) and bilaterality (126 cases,5.4%), 117 unilateral multifocal cases and 1819 unifocal cases were involved in the study. The collected patient data included age, gender, MTD, TTD, central lymph node metastasis (CLNM), lateral lymph node metastasis (LLNM), capsular invasion, gross extrathyroidal extension (gETE), and so on. The medical ethics committee of West China Hospital, Sichuan University approved the study. The patients in the study provided informed consent. All methods were carried out following relevant regulations and guidelines.

### Definition

In the study, MTD was referred to as either the primary tumor focus for unifocal PTC or the diameter of the greatest tumor focus in multifocal PTC. TTD was defined as the sum of each tumor lesion’s diameter within a lobe. Unilateral multifocality was defined as two or more lesions in the same lobe. CLNM was defined as tumor metastasis in level VI cervical lymph nodes. Based on the postoperative pathological diagnosis, LLNM was defined as tumor metastasis in lymph nodes of levels II, III, IV, and V in the neck region. Based on the postoperative pathology diagnosis, the capsular invasion was defined as a tumor involving the capsule but not penetrating it. gETE was characterized as the tumor penetrating the thyroid capsule, and invading surrounding tissues or organs, for example, esophagus, trachea and recurrent laryngeal nerve (RLN).

### Study design and grouping

A total of 1936 patients were involved in the study. First, the above data was divided into two groups according to the number of tumor lesions, the unilateral multifocal PTC group and the unifocal PTC group. PSM was performed based on age, sex, body mass index (BMI), nodular goiter (NG), Hashimoto’s thyroiditis (HT), capsule invasion, gETE, and TTD or MTD to minimize the effect of confounders on the outcomes between these groups. Then the data was divided into another two groups: the with CLNM group and the without CLNM group. PSM was performed based on age, sex, BMI, NG, HT, capsule invasion, gETE, TTD and MTD. Moreover, the data was divided according to have LLNM or not. And the same PSM as CLNM was performed.

### Surgical strategy

All patients enrolled in the study underwent total thyroidectomy. The central lymph nodes were routinely dissected. Patients found to have cervical lymph node metastases after undergoing fine-needle aspiration biopsy or preoperative imaging were candidates for lateral lymph node dissection. This diagnosis was verified by the intraoperative frozen sections.

### Statistical analysis

SPSS 26.0 software was applied to the data analysis. Continuous variables (confirmed by the Kolmogorov-Smirnov test) with non-normal distribution were presented as the median. Differences between continuous variables were examined using the Mann–Whitney U test. Categorical variables were presented as absolute value. Pearson’s χ2 test or Fisher exact test was used to investigate the heterogeneity between categorical variables. Univariate and multivariate logistic regression analyses were used to identify the risk factors for CLNM and LLNM. The receiver operating characteristic (ROC) curve was plotted and compared the area under the curve (AUC) values. R software (R Core Team, Version 4.1.2, Vienna, Austria) with the “matchit” package was used to perform the PSM. The nearest neighbor algorithm was used as the matching method, with the caliper value set to 0.02. A P value of < 0.05 was considered statistically significant.

## Results

### Comparison of baseline characteristics between the unilateral multifocal PTC group and the unifocal PTC group

A total of 1936 eligible patients were enrolled and categorized into the unilateral multifocal PTC group (n =117) and the unifocal PTC group (n = 1819). Demographic and clinicopathological characteristics of the patients were presented in **Table 1**. Before PSM, the unilateral multifocal PTC group had larger TTD (16.00[12.50-23.00]*vs*10.00[8.00-15.00], P<0.001). CLNM (62.4%*vs*48.9%, P=0.005) and LLNM (31.6%*vs*20.2%, P=0.003) were reported more frequently in the unilateral multifocal PTC group than in the unifocal PTC group. The patients with unilateral multifocal PTC were more likely to have NG (66.7%*vs*52.9%, P=0.009).

**Table 1:** Comparison of baseline characteristics between the unilateral multifocal PTC group and the unifocal PTC group.

		Before PSM		After 1:4 PSM (MTD)		After 1:4 PSM (TTD)	
	Unilateral Multifocal PTC (N=117)(%)	Unifocal PTC(N=1819) (%)	P	Unifocal PTC(N=468) (%)	P	Unifocal PTC(N=468) (%)	P
Age			0.694		0.457		0.439
<55	98(83.8)	1548(85.1)		378(80.8)		405(86.5)	
≥55	19(16.2)	271(14.9)		90(19.2)		63(13.5)	
Sex			0.705	(0)	0.780	(0)	0.684
Female	84(71.8)	1335(73.4)		342(73.1)		327(69.9)	
Male	33(28.2)	484(26.6)		126(26.9)		141(30.1)	
BMI	23.32(21.10-25.39)	23.07(20.81-25.10)	0.305	23.32(21.02-25.20)	0.789	23.32(20.96-25.39)	0.885
NG	78(66.7)	963(52.9)	**0.009**	319(68.2)	0.757	(0)	0.794
HT	26(22.2)	393(21.6)	0.913	110(23.5)	0.769	131(28)	0.208
Capsule invasion	33(28.2)	561(30.8)	0.549	152(32.5)	0.374	161(34.4)	0.203
gETE							
Esophageal	2(1.7)	29(1.6)	1	6(1.3)	0.663	8(1.7)	1
Tracheal	5(4.3)	46(2.5)	0.366	9(1.9)	0.170	12(2.6)	0.325
RLN	9(7.7)	123(6.8)	0.699	28(6)	0.497	35(7.5)	0.938
MTD	10.00(7.00-15.00)	10.00(8.00-15.00)	0.050	10.00(8.00-15.00)	0.587	15.00(10.00-21.00)	0.000
TTD	16.00(12.50-23.00)	10.00(8.00-15.00)	0.000	16.00(12.50-23.00)	0.000	15.00(10.00-21.00)	0.164
CLNM	73(62.4)	890(48.9)	0.005	204(43.6)	0.000	268(57.3)	0.314
LLNM	37(31.6)	368(20.2)	0.003	70(15)	0.000	116(24.8)	0.132
AJCC stage			0.202		0.049		0.354
I-II	112(95.7)	1777(97.7)		462(98.7)		456(97.4)	
III-IV	5(4.3)	42(2.3)		6(1.3)		12(2.6)	

1:4 PSM (MTD): PSM was performed basing on age, sex, BMI, NG, HT, capsule invasion, gETE and MTD.

1:4 PSM (TTD): PSM was performed basing on age, sex, BMI, NG, HT, capsule invasion, gETE and TTD.

BMI, Body mass index; NG, nodular goiter; HT, Hashimoto’s thyroiditis; RLN, recurrent laryngeal nerve; gETE, gross extrathyroidal extension; MTD, maximum tumor diameter; TTD, total tumor diameter; CLNM, central lymph node metastasis; LLNM, lateral lymph node metastasis; AJCC, American Joint Committee on Cancer.

The bold values: emphasize the p value<0.05.

After PSM was performed basing on age, sex, BMI, NG, HT, capsule invasion, gETE and MTD, significant differences between the unilateral multifocal PTC group and the unifocal PTC group in the rate of CLNM (62.4%*vs*43.6%, P<0.001) and LLNM (31.6%VS15.0%, P<0.001) still existed. And the unilateral multifocal PTC group had a higher proportion with AJCC stage III-IV (4.3%*vs*2.3%, P=0.049). After PSM was performed basing on age, sex, BMI, NG, HT, capsule invasion, gETE and TTD, differences between the unilateral multifocal PTC group and the unifocal PTC group disappeared in all characteristics except MTD (10.00[8.00-15.00]*vs*15.00[10.00-21.00], P<0.001). (**Table 1**).

### Comparison of baseline characteristics between the without LNM (lymph node metastasis) group and the with LNM group

**Table 2** listed the demographic and clinicopathological traits of the patients. CLNM occurred more frequently in male patients (30.9%*vs*22.5%, P<0.001), patients aged less than 55 years (87.9% *vs* 82.2%, P = 0.001), patients with lower BMI (22.89[20.70-25.00]*vs* 23.24[20.97-25.20], P<0.001). NG was reported less frequently in the With CLNM group (47.8%*vs*59.7%, P<0.001). While unilateral multifocality was reported more frequently in the With CLNM group (7.6%vs4.5%, P=0.005). There was a higher proportion of gETE in the With CLNM group (Esophageal 2.6% *vs*0.6%, P=0.001; Tracheal 3.7%*vs*1.5%, P=0.003; RLN 9.2%*vs*4.4%, P<0.001). The group with CLNM had larger MTD (13.00[10.00-20.00]*vs*10.00[7.00-14.00], P<0.001) and TTD (14.00[10.00-20.00]*vs*10.00[7.00-15.00], P<0.001). After PSM was performed basing on age, sex, BMI, NG, HT, capsule invasion, gETE, MTD and TTD, differences between the Without CLNM group and With CLNM group in the incidence of unilateral multifocality disappeared.

**Table 2:** Comparison of baseline characteristics between the Without CLNM group and the With CLNM group.

	Before PSM		P	After 1:1PSM		P
	Without CLNM(N=973) (%)	With CLNM(N=963) (%)		Without CLNM(N=713) (%)	With CLNM(N=713) (%)	
Age			**0.001**			0.141
<55	800(82.2)	846(87.9)		637(89.3)	619(86.8)	
≥55	173(17.8)	117(12.1)		76(10.7)	94(13.2)	
Sex			**0.000**			0.904
Female	754(77.5)	665(69.1)		523(73.4)	525(73.6)	
Male	219(22.5)	298(30.9)		190(26.6)	188(26.4)	
BMI	23.24(20.97-25.20)	22.89(20.70-25.00)	**0.000**	23.19(20.81-25.11)	22.86(20.70-24.84)	0.173
NG	581(59.7)	460(47.8)	**0.000**	370(51.9)	379(53.2)	0.633
HT	199(20.5)	220(22.8)	0.215	173(24.3)	120(16.8)	0.853
Capsule invasion	284(29.2)	310(32.2)	0.152	232(32.5)	226(31.7)	0.734
gETE						
Esophageal	6(0.6)	25(2.6)	**0.001**	6(0.8)	8(1.1)	0.591
Tracheal	15(1.5)	36(3.7)	**0.003**	15(2.1)	16(2.2)	0.856
RLN	43(4.4)	89(9.2)	**0.000**	39(5.5)	37(5.2)	0.814
MTD	10.00(7.00-14.00)	13.00(10.00-20.00)	**0.000**	10.00(8.00-15.00)	10.00(8.00-15.00)	0.432
TTD	10.00(7.00-15.00)	14.00(10.00-20.00)	**0.000**	10.00(8.00-15.00)	10.00(8.50-15.00)	0.227
Unilateral Multifocality	44(4.5)	73(7.6)	**0.005**	37(5.2)	46(6.5)	0.366

1:1PSM: PSM was performed basing on age, sex, BMI, NG, HT, capsule invasion, gETE, MTD and TTD.

BMI, Body mass index; NG, nodular goiter; HT, Hashimoto’s thyroiditis; RLN, recurrent laryngeal nerve; gETE, gross extrathyroidal extension; MTD, maximum tumor diameter; TTD, total tumor diameter.

The bold values: emphasize the p value<0.05.

Male patients (32.3%*vs*25.2%, P=0.004), patients without NG (43.7%*vs*56.4%, P<0.001), patients with gETE (Esophageal 5.2% *vs*0.7%, P<0.001; Tracheal 6.7%*vs*1.6%, P<0.001; RLN 14.1%*vs*4.9%, P<0.001), patients with larger MTD (15.00[10.00-25.00]*vs* 10.00[7.00-15.00], P<0.001) or TTD (16.00[10.00-25.00]*vs* 10.00[8.00-15.00], P<0.001), patients with unilateral multifocal PTC (9.1%*vs*5.2%, P=0.003) were more likely to have LLNM (**Table 3**). After PSM was performed basing on age, sex, BMI, NG, HT, capsule invasion, gETE, MTD and TTD, differences in the incidence of unilateral multifocality between the groups With and Without LLNM vanished.

**Table 3:** Comparison of baseline characteristics between the Without LLNM group and the With LLNM group.

	Before PSM			After 1:1PSM		
	Without LLNM(N=1531) (%)	With LLNM(N=405) (%)	P	Without LLNM(N=367) (%)	With LLNM(N=367) (%)	P
Age			0.958			0.915
<55	1302(85)	344(84.9)		315(85.8)	316(86.1)	
≥55	229(15)	61(15.1)		52(14.2)	51(13.9)	
Sex			**0.004**			0.431
Female	1145(74.8)	274(67.7)		242(65.9)	252(68.7)	
Male	386(25.2)	131(32.3)		125(34.1)	115(31.3)	
BMI	23.12(20.81-25.10)	23.07(20.96-25.20)	0.820	23.32(20.96-25.24)	23.03(20.76-25.10)	0.161
NG	864(56.4)	177(43.7)	**0.000**	157(42.8)	165(45)	0.552
HT	337(22)	82(20.2)	0.570	68(18.5)	79(21.5)	0.310
Capsule invasion	463(30.2)	131(32.3)	0.431	136(37.1)	124(33.8)	0.354
gETE						
Esophageal	10(0.7)	21(5.2)	**0.000**	9(2.5)	9(2.5)	1.000
Tracheal	24(1.6)	27(6.7)	**0.000**	16(4.4)	18(4.9)	0.725
RLN	75(4.9)	57(14.1)	**0.000**	44(12)	37(10.1)	0.410
MTD	10.00(7.00-15.00)	15.00(10.00-25.00)	**0.000**	15.00(10.00-20.00)	15.00(10.00-21.00)	0.742
TTD	10.00(8.00-15.00)	16.00(10.00-25.00)	**0.000**	15.00(10.00-22.00)	15.00(10.00-24.00)	0.545
Unilateral Multifocality	80(5.2)	37(9.1)	**0.003**	26(7.1)	34(9.3)	0.281

1:1PSM: PSM was performed basing on age, sex, BMI, NG, HT, capsule invasion, gETE, MTD and TTD.

BMI, Body mass index; NG, nodular goiter; HT, Hashimoto’s thyroiditis; RLN, recurrent laryngeal nerve; gETE, gross extrathyroidal extension; MTD, maximum tumor diameter; TTD, total tumor diameter.

The bold values: emphasize the p value<0.05.

### Risk factors for CLNM and LLNM

We used both univariate and multivariate analyses to obtain the risk factors for CLNM and LLNM. Univariate analysis identified 8 variables (Sex, Age, MTD, TTD, Esophageal extension, Tracheal extension, RLN extension and Unilateral multifocality) linked with CLNM, but the relationship among unilateral multifocality and CLNM was lost after PSM was performed basing on age, sex, BMI, NG, HT, capsule invasion, gETE, MTD and TTD (**Table 4**). Univariate regression analysis indicated that LLNM was significantly associated with sex, MTD, TTD, unilateral multifocality, esophageal extension, tracheal extension and RLN extension. The correlation between unilateral multifocality and LLNM was no longer present after PSM was performed basing on age, sex, BMI, NG, HT, capsule invasion, gETE, MTD and TTD (**Table 4**).

**Table 4:** Univariate logistic regression analysis of the risk factors for the CLNM and LLNM.

Variables	Before PSM		P	After 1;1 PSM		P
	OR	[95%CI]		OR	[95%CI]	
CLNM						
Sex (*vs* male)	0.648	0.529-0.794	0.000			
Age (*vs*≥55)	1.564	1.213-2.015	0.001			
MTD	1.080	1.065-1.095	0.000			
TTD	1.081	1.067-1.096	0.000			
Unilateral Multifocality	1.732	1.178-2.546	0.005	1.260	0.807-1.968	0.310
gETE						
Esophageal	4.295	1.754-10.518	0.001			
Tracheal	2.48	1.349-4.560	0.003			
RLN	2.202	1.513-3.206	0.000			
LLNM						
Sex (*vs* male)	0.705	0.556-0.895	0.004			
MTD	1.082	1.068-1.095	0.000			
TTD	1.083	1.070-1.097	0.000			
Unilateral Multifocality	1.824	1.215-2.737	0.004	1.339	0.786-2.281	0.282
gETE						
Esophageal	8.318	3.885-17.810	0.000			
Tracheal	4.485	2.559-7.862	0.000			
RLN	3.18	2.210-4.575	0.000			

1:1PSM: PSM was performed basing on age, sex, BMI, NG, HT, capsule invasion, gETE, MTD and TTD.

RLN, recurrent laryngeal nerve; gETE, gross extrathyroidal extension; MTD, maximum tumor diameter; TTD, total tumor diameter; CLNM, central lymph node metastasis; LLNM, lateral lymph node metastasis.

Multivariate analysis found 3 variables (Sex, Age, TTD), which were risk factors of CLNM, and 3 variables (TTD, Esophageal extension, Tracheal extension), which were risk factors of LLNM (**Table 5**).

**Table 5:** Multivariate logistic regression analysis of the risk factors for the CLNM and LLNM.

Variables	OR	[95%CI]	P
CLNM			
Sex(*vs* male)	0.713	0.575-0.883	0.002
Age(*vs*≥55)	1.803	1.371-2.371	0.000
TTD	1.106	1.041-1.175	0.001
LLNM			
TTD	1.098	1.037-1.163	0.001
gETE			
Esophageal	2.849	1.157-7.014	0.023
Tracheal	1.997	1.006-3.962	0.048

1:1PSM: PSM was performed basing on age, sex, BMI, NG, HT, capsule invasion, gETE, MTD and TTD.

gETE, gross extrathyroidal extension; TTD, total tumor diameter; CLNM, central lymph node metastasis; LLNM, lateral lymph node metastasis.

### Comparison of prediction efficiency of TTD and MTD for CLNM and LLNM

**Figure 1:**
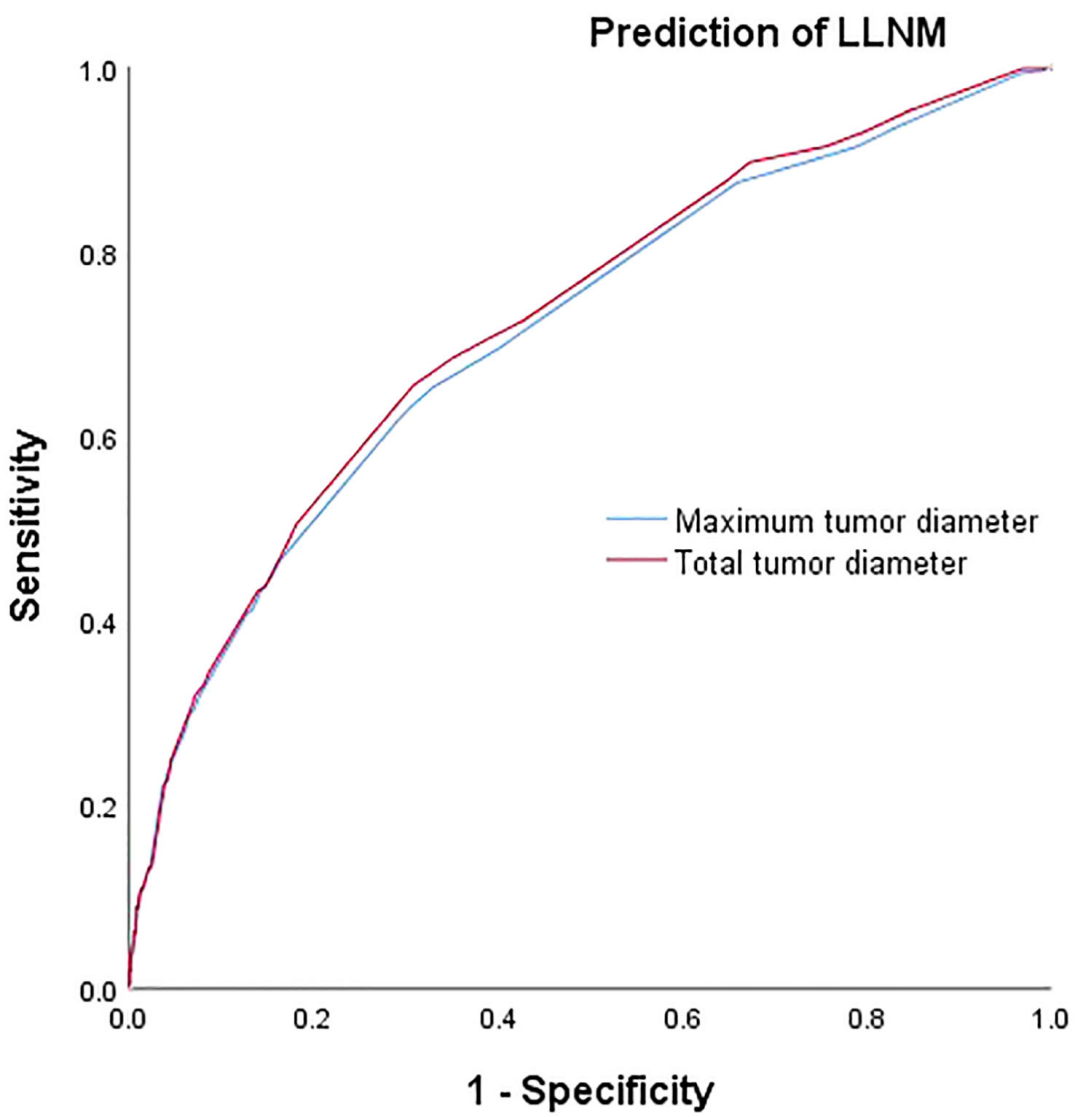
Comparison of prediction efficiency of TTD and MTD for CLNM. AUC values of 0.660(MTD) and 0.669(TTD) differed statistically (P=0.005). MTD, maximum tumor diameter; TTD, total tumor diameter; CLNM, central lymph node metastasis.

**Figure 2:**
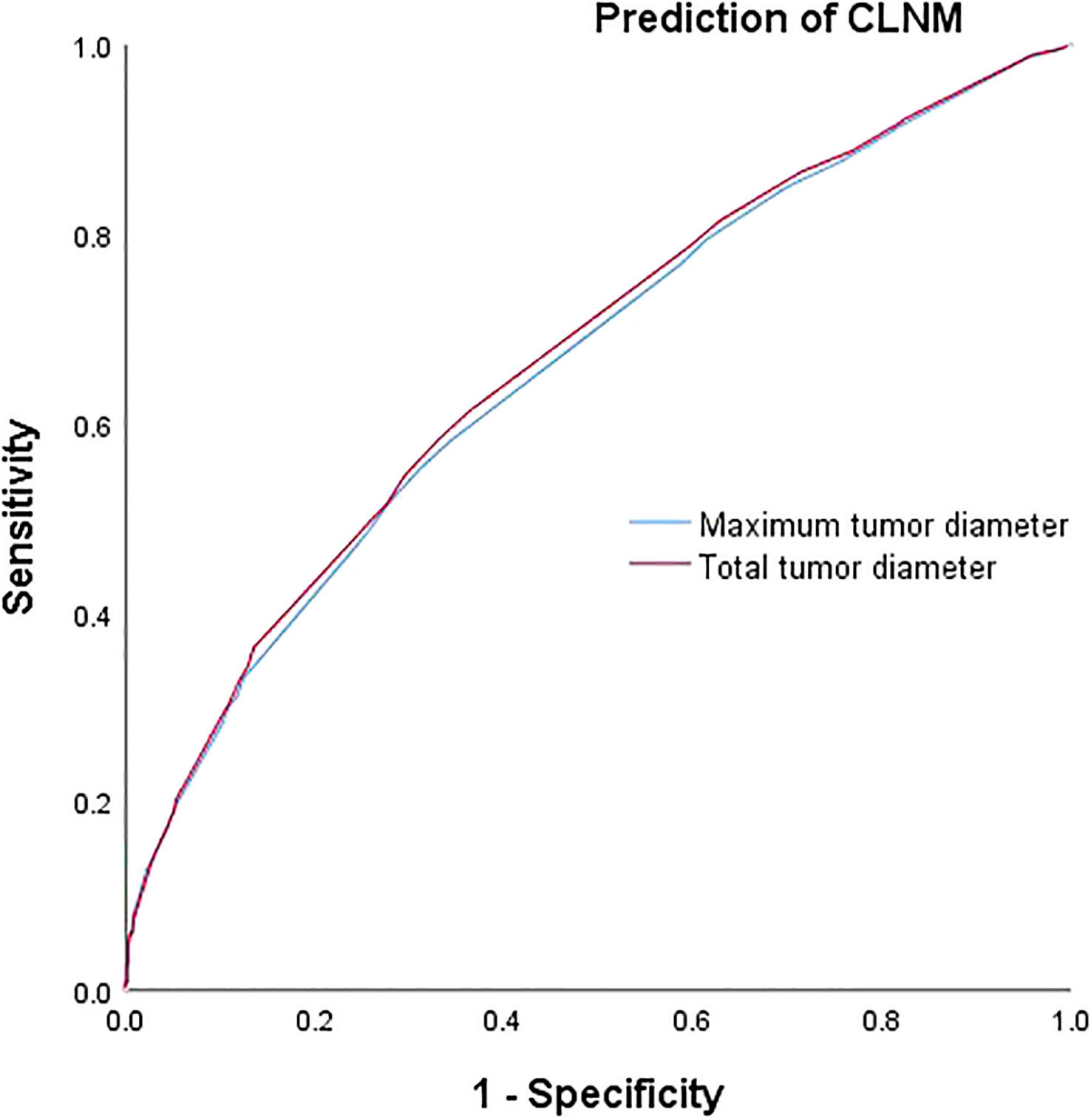
Comparison of prediction efficiency of TTD and MTD for LLNM. AUC values of 0.712(MTD) and 0.723(TTD) differed statistically (P=0.011). MTD, maximum tumor diameter; TTD, total tumor diameter; LLNM, lateral lymph node metastasis.

The respective calculated AUC values of 0.660(MTD) and 0.669(TTD) were statistically significantly different (P=0.005) (**Table 6**), suggesting that TTD may be a better predictor of CLNM for unilateral multifocal PTC (**Figure 1**). Meanwhile, the computed AUC values of 0.712(MTD) and 0.723(TTD), respectively, differed statistically(p=0.011) (**Table 6**), showing that for unilateral multifocal PTC, TTD may be a stronger predictor of LLNM (**Figure 2**).

**Table 6:** The diagnosis efficiency of LNM comparison between MTD and TTD.

Diagnostic indicators	Cutoff value	AUC	95%CI	P	Sensitivity	Specificity	Youden index
CLNM							
MTD	11.5	0.660	0.636-0.684	0.000	0.551	0.689	0.240
TTD	11.5	0.669	0.519-0.632	0.012	0.584	0.668	0.252
LLNM							
MTD	13.5	0.712	0.683-0.742	0.000	0.630	0.696	0.326
TTD	14.5	0.723	0.695-0.752	0.000	0.654	0.692	0.346

MTD, maximum tumor diameter; TTD, total tumor diameter; LNM, lymph node metastasis; CLNM, central lymph node metastasis; LLNM, lateral lymph node metastasis.

## Discussion

The impact of TTD in multifocal tumors has been reported on diseases like breast cancer and kidney cancer (13–15). Additionally, TTD’s significance in thyroid cancer has been documented in prior researches. They found that TTD was associated with lymph node metastasis and may represent the tumor biological traits and aggressiveness more accurately than MTD (9, 11, 12, 16). However, the researchers treated unilateral multifocality and bilaterality as the same multifocality types. The current study used a PSM method to investigate the association between TTD and clinicopathological characteristics in unilateral multifocal PTC based on thyroid cancer patients in our center.

The AJCC TNM classification determines the primary tumor (T) stage according to the largest tumor size and ETE (17), even in the multifocal PTC. The impact of other smaller tumor foci on aggressiveness has not been well investigated. Evaluating the largest tumor size and neglecting the smaller foci in multifocal tumors may underestimate the clinicopathologic aspects of malignancies, impacting tumor staging and treatment. In our study, we did two different types of PSM, matching the MTD and TTD respectively, to explore which diameter could represent the tumor biological characteristics and aggressiveness more accurately. We found that the lymph node metastasis rate had significant differences between unilateral multifocal PTC and unifocal PTC after PSM matching the MTD. However, no difference was found after PSM matching the TTD. In other words, the tumor biological traits of the unilateral multifocal PTC were similar to the unifocal PTC with approximate TTD. This result was identical to the previous study (11), which mainly focused on papillary thyroid microcarcinoma (PTMC), showing that micro-PTC tended to behave like the macro-PTC once the TTD exceeded 1.0 cm.

LNM was common among PTC patients. According to earlier research, LNM was present in 30% to 90% of PTC patients, particularly in the central region (18–20), which was one of the major factors that contributed to postoperative recurrence. Although reoperation could still result in a favorable prognosis, patients were more probably to suffer surgical complications (21, 22). Therefore, it was crucial for the patient’s overall prognosis that the lymph nodes were managed at the initial surgery. Because the unique anatomical position limited the diagnostic ability of the current imaging techniques (23), the clinical lymph nodes negative (cN0) was unreliable. Whether prophylactic central lymph nodes dissection (PCLND) was appropriate for cN0 PTC patients was still up for debate. PCLND at the time of the initial surgery, according to Guo et al. (21, 24), may have advantages, including avoiding the significant risk of long-term hypoparathyroidism and RLN damage associated with repeat surgery. Yoon et al. (25), on the other hand, came to the conclusion that the chance of developing permanent hypocalcemia may rise and parathyroid hormone levels may be dramatically lowered following PCLND. In order to direct therapeutic decision-making, it was helpful to identify high-risk variables for LNM. In our study, TTD was significantly correlated with CLNM and LLNM. Also, the ability of TTD to predict CLNM and LLNM was much better than that of MTD in PTC, showing that TTD might be a better predictor of LNM for unilateral multifocal PTC.

In our data, 62.4% of unilateral multifocal PTC presented with CLNM and 31.6% presented with LLNM, significantly higher than unifocal PTC (48.9%&20.2%). Other researches showed the same results (26, 27). In a report by Lombardi et al., multifocal illness increased cervical lymph node recurrence 17.9-fold (28). In another study, the rate of LNM for patients with multifocal PTCs was 55.6%, while it was only 28.6% for those with unifocal illnesses (29). However, the association between LNM and multifocality remained controversial. According to certain studies, multifocality was not independently related to LNM (30). Many other studies have focused on the risk factors of LNM for multifocal PTC patients. For example, Zheng WH proposed that multifocality should be evaluated when selecting patients for PCLND (31). Some reports showed that multifocality was an independent risk factor for CLNM and LLNM (32, 33). As found in previous studies (33–36), our univariate analyses revealed an association between multifocality and LNM. However, on multivariate adjustment for classic clinicopathological risk factors, including MTD and TTD, the association between unilateral multifocality and LNM was lost in PTC. The result demonstrated that unilateral multifocality played no independent role in LNM.

Once, it was thought that multifocality in PTC was caused by the lymphatic spread of tumor cells from one malignant clone. This theory was backed by the fact that tumor cells were often found in lymphatic vessels and that lymph node metastases happened often. But others had the opposite ideas. Bansal et al. found that as many as 60% of multifocal PTCs may have multiple sources, and these origins were generally found in various lobes. The same mutation status was typically shared by multifocality within the same lobe, though (37, 38). A clinical research also showed that unilateral multifocality differed from bilaterality (5). Unlike earlier studies (5, 11), we only included unilateral multifocal PTC in our study to remove the distraction of different types of multifocality and used PSM method to provide an updated and more comprehensive assessment of the risk for unilateral multifocal PTC.

There were some limitations in the study. The inclusion of patients based on their characteristics resulting in a selection bias in this retrospective analysis. Despite the fact that we used PSM, the results could still be influenced by selection bias. Moreover, the participant sample of unilateral multifocal PTC was small. Our data could be hampered by the small sample size. Conducting more validation studies with a bigger cohort and long-term follow-up is necessary.

## Conclusion

In conclusion, the current study offers fresh evidence of the connection between TTD and PTC. TTD can more accurately reflect the biological characteristics and aggressiveness of unilateral multifocal PTC than MTD.

## Data availability statement

The original contributions presented in the study are included in the article/supplementary material. Further inquiries can be directed to the corresponding author.

## Ethics statement

The studies involving humans were approved by The medical ethics committee of West China Hospital, Sichuan University. The studies were conducted in accordance with the local legislation and institutional requirements.

## Author contributions

Author Contributions (I) Conception and design: A-PS, Z-JW (II) Administrative support: A-PS (III) Provision of study materials or patients: A-PS, Z-JW (IV) Collection and assembly of data: Z-JW, Z-WC, B-YX, HG (V) Data analysis and interpretation: Z-JW, MA. All authors contributed to the article and approved the submitted version.
